# Youngest Baby on Record: Is It?

**Published:** 1889-07

**Authors:** Sam I. Fox

**Affiliations:** Plantersville


### YOUNGEST MOTHER ON RECORD. IS IT?

PLANTERSVILLE, July 8, 1889.

Editor Daniel's Texas Medical Journal :

I was called to see a case on June 30th. When I reached the house and went in the room, I saw a very small girl on the bed, and her mother said to me, "Doctor, my daughter miscarried on yesterday, and we wish you to take the afterbirth," which I did, and she is now doing well. When I was through, I asked how old she was, and father and mother both told me she was hardly twelve years old.

Now, I wish you to publish this case, and see whether there is a younger mother on record or not. Her father is a full blooded negro, and her mother is a mulatto. Please report the case, and let us see if there is a younger mother on record.

Yours respectfully,

SAM I. FOX.

